# Detection of Murine Astrovirus and *Myocoptes musculinus* in individually ventilated caging systems: Investigations to expose suitable detection methods for routine hygienic monitoring

**DOI:** 10.1371/journal.pone.0221118

**Published:** 2019-08-13

**Authors:** Carolin Körner, Manuel Miller, Markus Brielmeier

**Affiliations:** Helmholtz Zentrum München—German Research Center for Environmental Health, Research Unit Comparative Medicine, Neuherberg, Germany; University of Illinois at Urbana-Champaign, UNITED STATES

## Abstract

Murine Astrovirus is one of the most prevalent viral agents in laboratory rodent facilities worldwide, but its influence on biomedical research results is poorly examined. Due to possible influence on research results and high seroprevalence rates in mice, it appears useful to include this virus into routine health monitoring programs. In order to establish exhaust air particle PCR as a reliable detection method for Murine Astrovirus infections in mice kept in individually ventilated cages (IVC) and compare the method to sentinel mice monitoring regarding reproducibility and detection limit, we conducted a study with defined Murine Astrovirus cage prevalence. In parallel, the efficacy of both detection strategies (soiled-bedding sentinel (SBS) and exhaust air dust (EAD) analysis) was tested for *Myocoptes musculinus*. The fur mite was used as a reference organism during the whole study period to ensure the validity of this method. Because some publications already demonstrated successful detection of several pathogens, including murine fur mite species, via EAP-PCR. Detection of Murine Astrovirus infections at low prevalence is possible with both methods tested. Detection by exhaust air particles (EAP) is faster, more sensitive and more reliable compared to soiled bedding sentinels (SBS). Exhaust air particle PCR also detected the reference organism *Myocoptes musculinus*, which was not detected at all by sentinel mice, not even by high sensitivity fur swab qPCR. In conclusion, Murine Astrovirus can be detected by both exhaust air particle PCR and soiled bedding sentinels. We recommend exhaust air particle PCR as the better detection technique for Murine Astrovirus, because it is more reliable. Environmental samples are the method of choice for detection of *Myocoptes musculinus* because relying on soiled bedding sentinels harbors a big risk of missing existing infestations.

## Introduction

Astroviruses (Family *Astroviridae*) represent a huge and diverse phylogenetic group infecting mammals and birds [1–3] first discovered in the mid-1970s in children with gastrointestinal illness. Due to high seroprevalence rates of Human Astroviruses (HAstV) (in early childhood up to 90% of the population have developed antibodies against HAstV-1 [4–6]) and their worldwide occurrence, there is great interest in characterizing these viruses in more detail, because even today astroviruses are still among the least studied enteric RNA viruses. [1] In many cases, astrovirus infections are limited to the gastrointestinal tract. [6,7] However, depending on the immune status and species of the host, severe extragastrointestinal or asymptomatic courses have also been detected. [8–18] Biology and pathogenesis of astroviruses are still largely unexplored due to the great genetic variety and broad host spectrum. [1,19] In mice the typical star-shaped appearance that gives the name to astrovirus family was first recognized by electron microscopy in gut contents in 1985. [20] Based on real time PCR analysis, a worldwide distribution of Murine Astroviruses (MuAstV) was assumed, both in biomedical research institutions and commercial breeders. [21,22] A first sero-epidemiological study verified these presumptions based on a multiplex MuAstV serology assay. [23]

Hence, MuAstV is one of the most common viral agents in laboratory rodent facilities. Both immunocompetent and immunodeficient mouse strains are affected. However, MuAstV apparently does not cause any clinical signs in mice. But its influence on biomedical research results is poorly examined. Merely, Yokoyama et al. [24] demonstrated that the adaptive and innate immune system is affected. [1] The former one is important in limiting virus replication as it has also been shown for human astroviruses. [8,25] An influence of astrovirus infections on the microbiome of bats has been recently demonstrated. [26] It is assumed that murine microbiome studies might also be affected. [1] All in all it appears useful to include MuAstV to routine health monitoring and be aware of its possible influence on research results. Furthermore in 2012, it was shown that laboratory mice are suitable mammalian models to improve our understanding of astrovirus infection and virus host interaction. [24] In this context the detection of this agent in research mice is essential.

Study results on detection strategies of Murine Astrovirus are only sparsely available. Compton et al. [27] showed, that MuAstV is readily transferable to sentinels via soiled bedding and storing feces for up to 3 weeks did not affect the infectivity of the virus. Likewise, MuAstV was detected in the exhaust air dust, but not with the same sensitivity at all exhaust air sections of the rack. [27] Exhaust air particle PCR (EAP-PCR) has already proven to be a suitable and sensitive detection method for the detection of other pathogens. [28–32] In order to establish EAP-PCR as a reliable detection method for MuAstV in individually ventilated caging systems (IVCs) and to compare it to sentinel monitoring regarding reproducibility and detection limit, we conducted a study with defined MuAstV cage prevalence.

For *Myocoptes musculinus (M*. *musculinus)*, however, both detection strategies (soiled-bedding sentinels (SBS) and exhaust air dust (EAD) analysis) have already been tested for their efficacy. Therefore, we decided to use the fur mite at very low prevalence as a reference organism during the whole study period to ensure the validity of this method. Because some publications already demonstrated successful detection of several pathogens, including murine fur mite species, via EAP-PCR, yet at unknown prevalence. [28,30,33–37]

## Materials and methods

### Study design

**Fig 1:**
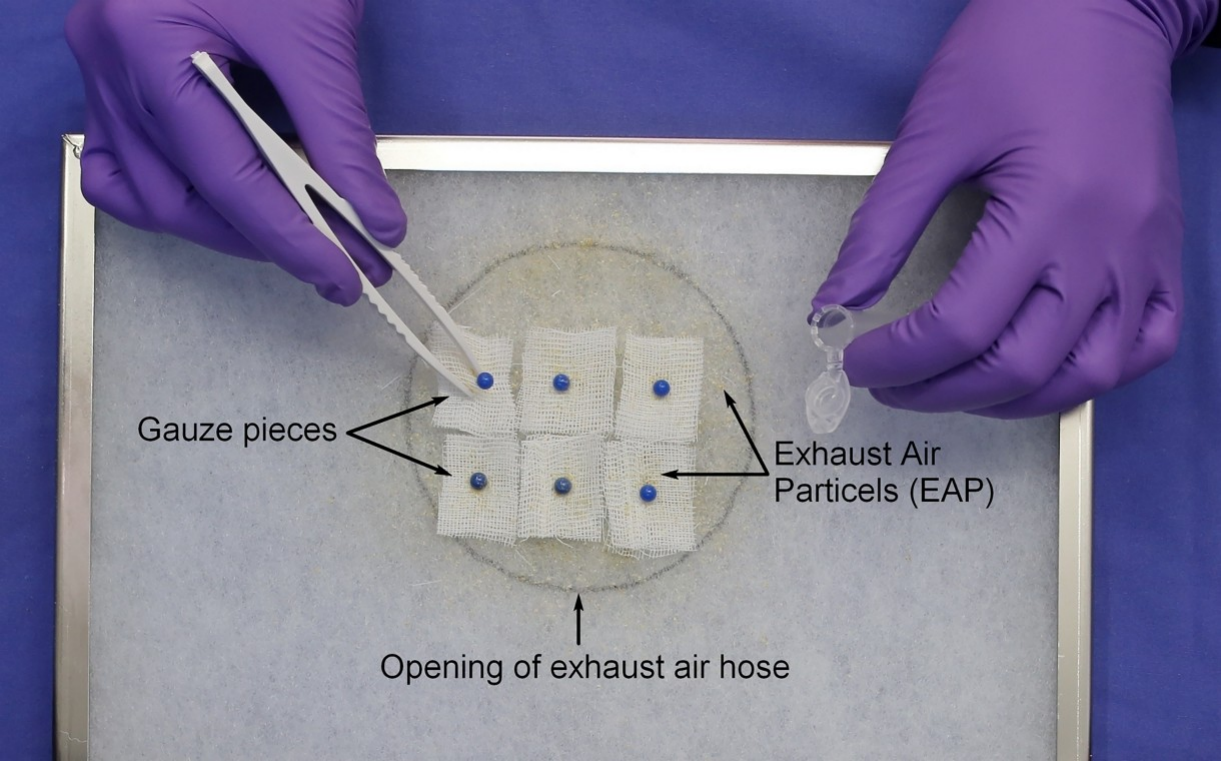
Schematic representation of EAP sampling. The black circle marks the position of gauze pieces attached to the prefilter directly above the opening of the exhaust-air hose in the ventilation unit. Gauze pieces were attached within this area using pins and were removed weekly with disposable forceps.

The study design was adapted slightly to the one used in our study on *Pasteurella pneumotropica* detection [28]. In short, in order to compare the detection of MuAstV and *M*. *musculinus* by soiled-bedding sentinels (SBS) or exhaust air particle (EAP) PCR, a colony of MuAstV- and *M*. *musculinus*-negative mice (59 cages in total) was kept together with a defined number of MuAstV-infected (3 cages of 5 mice each) and *M*. *musculinus*-infested mice (1 cage of 5 mice) in an IVC rack. The rack system used in this study includes 63 cages arranged in 9 rows (A-I) and 7 columns (1–7). The cages containing the infected mice were mounted at the same location in the middle row in central position of the rack in each repetition (E3-E6). In order to maintain this defined prevalence, the positive animals were also tested for each other's pathogens. MuAstV-positive animals were tested for *M*. *musculinus* by fur swabs in each experimental repetition and vice versa, the *M*. *musculinus*-positive animals were tested for MuAstV by fecal pellets. EAP samples and sentinels were tested for MuAstV and *M*. *musculinus* at several time points. The experiment was repeated 7 times for reasons of statistical significance. Low cage prevalence was chosen for *M*. *musculinus* based on pilot experiments. At the beginning of each repetition, baseline tests were performed to ensure the status of the negative colony and that all cage equipment is free of MuAstV and *M*. *musculinus* nucleic acids. All cages, cage supplies, rack systems and tubes were washed thoroughly in a tunnel or a rack washer to remove residual nucleic acids and autoclaved. For the same reason the air handling unit was cleaned, disinfected and the prefilter was changed before each experimental repetition. Baseline testing included on the one hand EAP-PCR of gauze pieces attached to the prefilter directly above the opening of the exhaust-air hose in the ventilation unit (Fig 1). After one week, gauze pieces were removed and analyzed by qPCR to confirm absence of MuAstV and *M*. *musculinus* nucleic acids in the exhaust air of the test rack. On the other hand, the status of the negative colony was confirmed prior to each experimental repetition by qPCR analysis of fecal pellets (10 pellets/cage, for MuAstV) and fur swabs (1 swab per cage, pooled to 3 swabs per sample, for *M*. *musculinus*). Pooled fecal pellets and fur swabs were tested in house by qPCR assays for MuAstV [24] and *M*. *musculinus*.

For quality assurance purposes further samples were taken at the end of each experimental repetition and were sent for qPCR to a commercial diagnostic laboratory. Therefore, cage swabs (1 swab per cage, pooled to 10 swabs per sample) were taken from the negative colony as well as fecal pellets and fur swabs from sentinels (after 12 weeks).

After the baseline tests confirmed absence of residual nucleic acids on the rack system and the status of the negative mouse colony (= end of the baseline test week), MuAstV-infected and *M*. *musculinus*-infested mice (4 cages in total) were added to the test rack and new gauze pieces were attached to the prefilter. For the whole testing period of twelve weeks cages were changed weekly and sentinel mice received 50% soiled bedding from the negative and positive colony mixed with 50% fresh bedding. The soiled bedding sentinels were maintained in a separate IVC rack (equal model in the same room) to avoid an increase in prevalence of infected or infested mice in the study rack. Sentinel mice were tested for MuAstV at 2, 4, 6, 8, 10 and 12 weeks and for *M*. *musculinus* at 6 and 12 weeks after they received soiled bedding for the first time. Gauze pieces for EAP-PCR were taken and analyzed for the two agents weekly and fresh gauze pieces were mounted on the prefilter. Based on the fact that shedding of MuAstV via feces is temporary, positive animals were checked weekly via fecal PCR. *M*. *musculinus*-infested mice were retested at the beginning and end of every repetition, since *M*. *musculinus* lives permanently at the host [38] and rapid elimination is not expected. An experimental round was finished after 2 consecutive positive gauze PCRs for MuAstV. Whereas soiled-bedding sentinels of this experimental repetition continuously received soiled bedding for a total of 12 weeks.

### Animal housing

All mice were housed in IVC systems (GM 500, SealSafe Plus, Tecniplast, Buggugiate, Italy) under SPF conditions with a maximum density of 5 adult animals per cage. All racks, cages and cage supplies were cleaned and autoclaved prior to use. The mice were kept at the following environmental conditions; 12:12-h light:dark cycle, 20–24°C, 45–65% relative humidity. The ventilation units ran at 60 air changes per hour at positive pressure (15–22 Pa). Autoclaved wood chips (SAFE Select Fine, J Rettenmaier & Söhne GmbH, Rosenberg, Germany) and nesting material (SAFE Crinklets natural, J Rettenmaier & Söhne GmbH) were used as bedding and environmental enrichment. Sterile-filtered tap water and irradiated standard rodent chow (Altromin 1314, Altromin Spezialfutter, Lage, Germany) were available ad libitum. To access the animal husbandry complete change of clothes and shoes, passing an air shower and wearing sterilized protective clothing and gloves, mouth protection and hair net was mandatory. Cage changes and sampling were performed under a HEPA-filtered cage changing station. Routine health monitoring was performed quarterly for all FELASA-listed agents [39] based on EAP-PCR [30].

### Mice

All mice used were excess mice obtained from breeding colonies at our facility. MuAstV- and *M*. *musculinus-*negative colony consisted of immunocompetent AVM:ICR mice of both sexes and different age (3–7 months old). Mice were kept in stable groups of compatible animals (2-5 per cage) separated by gender. Female AVM:ICR mice (age app. 2 months) were kept as soiled-bedding sentinels in groups of 3 animals per cage. Negative colony and sentinels were obtained from an SOPF breeding barrier repeatedly tested negative during routine health monitoring for all FELASA-listed agents [39] including *M*. *musculinus* and MuAstV. MuAstV-positive mice were generated by co-housing naïve AVM:ICR of both sexes and different ages (about 4 weeks–6 months) with MuAstV positive mice. MuAstV infection and virus shedding was confirmed weekly per MuAstV-specific qPCR of fecal pellets (1-3 pellets/animal). MuAstV-infected mice were kept in groups of 5 animals per cage, separated by gender. The *M*. *musculinus-*positive colony consisted of female immunocompetent C57BL/6N mice (age, 4–16 weeks). Their status of infestation was evaluated via fur swab qPCR analysis. *M*. *musculinus*-infested mice were kept in groups of 5 animals per cage, separated by gender. Mice showing clinical signs of illness were immediately removed from the study and replaced by tested and uninfected ones. These mice were killed by trained staff using cervical dislocation in accordance with animal welfare regulations. At the end of the experiment old mice were also euthanized by cervical dislocation. Younger mice were further used for educational and training purposes. The study was carried out in strict accordance with Directive 2010/63/EU and has been approved by the responsible authority (Regional Council of Upper Bavaria, Germany) under reference number: 55.2-1-54-2532.0-89-2016.

### MuAstV RNA extraction and cDNA synthesis

MuAstV-RNA was extracted using QIAamp viral RNA Mini kit (Qiagen, Valencia, CA). Fecal pellets were homogenized with 700 μL AVL buffer mixed with 7 μL Carrier-RNA/AVE by the help of a tissue homogenizer instrument (TissueLyzer LT, Qiagen, 5 min, 50 Hz) and stainless steel beads (1 bead per reaction, 7 mm, Qiagen) in a 2 mL microcentrifuge tube. Homogenates were centrifuged for 9 min at 8000 rpm (Eppendorf Centrifuge 5415 D). After sample preparation, 140 μL of the sample solution were pipetted into 560 μL AVL buffer and further processed according to the manufacturer´s protocol. Preparation of exhaust air dust samples differs slightly from above. Instead of homogenizing them with a tissue homogenizer instrument, they were shaken thoroughly in a thermomixer (Eppendorf Thermomixer comfort) with 1120 μL AVL buffer mixed with 11.2 μL Carrier-RNA/AVE at room temperature for 20 min. After incubation, 700 μL of the supernatant were transferred into a fresh 2 mL microcentrifuge tube. Process continues according to the manufacturer´s protocol. Synthesis of first strand cDNA of MuAstV-RNA templates was performed by using Thermo Scientific RevertAid RT Kit. For primer mix, 5 μL of total fecal RNA volume was mixed with 1 μL random hexamer primers and 6.5 μL nuclease free water resulting in a total volume of 12.5 μL. Vials were mixed gently, centrifuged briefly and incubated for 5 min at 65°C in a Thermocycler (T-1 Thermocycler, Biometra). After incubation at 4°C for 5 min, Mastermix consisting of 4 μL of 5x reaction buffer, 0.5 μL of RiboLock RNA Inhibitor (20 U/μL, effectively protects RNA from degradation at temperatures up to 55°C), 2 μL of 10 mM dNTP Mix and 1 μL of RevertAid Reverse Transcriptase (200 U/μL) were added to each sample to a total volume of 20 μL. For random hexamer primer synthesis, vials were incubated for 10 min at 25°C, followed by 60 min at 42°C and termination of the reaction for 10 min at 70°C. Appropriate reaction controls to verify the results were used for each run.

### *M*. *musculinus* DNA extraction

*M*. *musculinus*-DNA isolation from fur swabs and gauze pieces was accomplished using phenol/chloroform extraction method. Individual fur swabs, gauze pieces and pooled fur swabs were mixed with 400 μL, 500 μL, and 650 μL of lysis buffer (10 mM EDTA, 10 mM Tris-HCl pH 7.6, 0.5% SDS, 10 mM NaCl, and 0.3 mg/mL Proteinase K), respectively; and incubated for 30 min at 55°C in a thermomixer (Eppendorf Thermomix comfort) at 600 rpm. The dissolved DNA (300 μL) was transferred into a new 1,5 mL microcentrifuge tube. After adding the same amount of Roti phenol-chloroform-isoamylalkohol (Roth, Karlsruhe, Germany), the solution was vortexed and centrifuged for 6 min at room temperature at 13000 rpm. Subsequently, 200 μL of the upper aqueous phase were pipetted in a new microcentrifuge tube filled with 500 μL of NaCl-saturated 100% ethanol (15 μL 5 M NaCl/mL absolute EtOH) and centrifuged for 10 min at 4°C at 13000 rpm to precipitate the DNA. Afterwards supernatant was removed and DNA pellet was washed twice with 200 μL of 70% EtOH and centrifuged for 2 min at 4°C at 13000 rpm. Nucleases free water (25 mL) was used to dissolve the purified DNA pellet. Extracted DNA was not quantified prior usage in qPCR.

### Quantitative PCR analysis

MuAstV-specific quantitative PCR analysis was performed using primers and probe established by Yokoyama et al. 2012 [24] binding to an 80 bp region of ORF 1b in a RotorGene Q instrument (5-Plex HRM, Qiagen).

The sequences of the primers and probes are as follows: Forward 5’-TACATCGAGCGGGTGGTCGC-3’, Reverse 5’-GTGTCACTAACGCGCACCTTTTCA-3’, TaqMan probe 6FAM-TTTGGCATGTGGGTTAA-MBGNFQ. To quantify the amount of MuAstV in the samples examined, a triplicate plasmid DNA dilution series, ranging from 1 x 10^6^ to 1 x 10^1^ copies/μL was included in each repetition. In addition to detect contaminants in the reaction mix a no-template, reverse transcription, positive and negative control was added to each run. Data were analyzed with the RotorGene Q Software (version 2.3.1).

*M*. *musculinus* qPCR assay for specific amplification of a sequence within the cytochrome b gene was designed in house using the program Primer 3. *M*. *musculinus*-specific qPCR assay was performed using the following primers: Forward 5´-GCC TCTTCA ACA TCA ACC CC-3´ and Reverse 5´-TTA TAC AAA CTA CCC CAC CAA GT-3´ and a 6-FAM-BHQ-1 dual labeled fluorogenic probe 5´-TCT ACG TGC CAT TCC GTC CA-3´ in a Rotor gene Q instrument (5-Plex HRM, Qiagen). DNA template (2 μL) was added to a reaction mix consisting of 4 μL of 5x HOT FIREPol Probe qPCR Mix Plus (Solis BioDyne, Tartu, Estonia), 0.4 μL of each primer (5 μM), 0.4 μL probe (10 μM), and ultrapure water in a 18 μL total reaction volume. The following thermocycling conditions were used for amplification: initial denaturation at 95°C for 15 min, ensued by 40 cycles consisting of 15 s denaturation at 95°C and annealing-extension for 1 min at 60°C. To quantify the amount of *M*. *musculinus* in the samples examined, a plasmid was constructed by cloning the 111 bp qPCR amplicon into the pCR2.1-TOPO vector (TOPO TA Cloning kit, Invitrogen by Life technologies, Waltham, MA) according to the manufacturer´s instructions. A triplicate plasmid DNA dilution series ranging from 1 x 10^7^ to 1 x 10^1^ copies/μL was included in each repetition. In addition to detect contaminants in the reaction mix a no-template control, positive and negative control was added to each run. Data were analyzed by using RotorGene Q Software (version 2.3.1).

Sensitivity was determined to be 1 copy/μl. All samples resulting in more than 1 copy/μL were considered positive. Samples between 0 to 1 copy/μL were considered questionable and were retested; they were scored as positive when the copy number was again greater than 0. Otherwise they were considered negative.

## Results

### Comparison of EAP-PCR and SBS for detecting MuAstV infection in IVC-housed mouse colonies

#### Baseline tests

**Fig 2:**
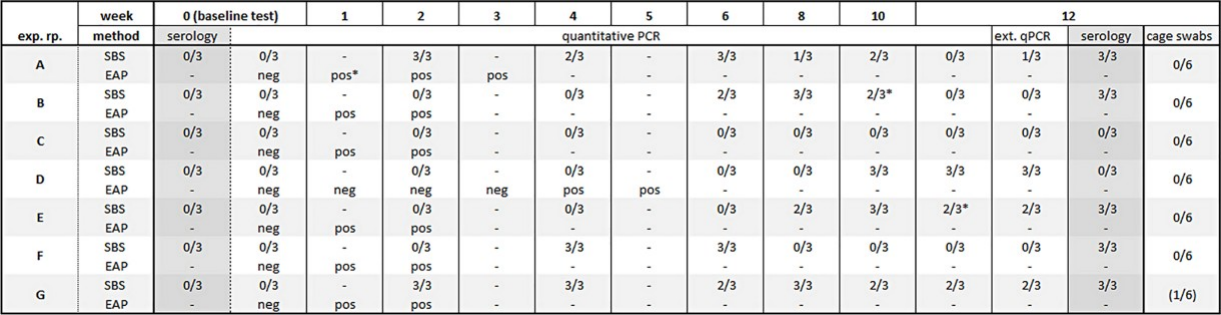
Comparison of qPCR and serology results for soiled-bedding sentinel (SBS) and Exhaust Air Particle (EAP) monitoring at known prevalence of MuAstV. MuAstV = Murine Astrovirus; Exp. rp. = Experimental repetition; SBS = Soiled bedding sentinels; EAP = Exhaust air particle; ext. qPCR = quantitative PCR performed at an external provider; pos = positive EAP qPCR result for MuAstV; neg = negative EAP qPCR result for MuAstV; numerals = number of positive Sentinels per number sentinels tested for qPCR or serology dash, not tested; asterisk, samples between 0–1 copy / μL.

At the start of each of seven experimental repetitions all SBS were serologically negative for MuAstV. An acute infection was ruled out through qPCR of individual fecal pellets of all SBS. No residual nucleic acids (MuAstV RNA) were detected in the IVC-rack by qPCR analysis of gauze pieces exposed to the rack with the negative colony for one week. In summary, all baseline tests were negative (Fig 2).

#### Soiled bedding sentinels

In 6 of 7 repetitions SBS got infected with MuAstV by contact with soiled bedding as shown by positive fecal qPCR at 2, 6, 10, 8, 4, 2 weeks after exposure in repetitions A, B, D, E, F, and G respectively (Fig 2). In repetition C, SBS fecal PCR was negative until the end of the surveillance period (weeks 12). SBS seroconverted to MuAstV in repetitions A, B, E, F and G, but not in C and D, although in D, the feces of the SBS were PCR positive in weeks 10 and 12. Durations of fecal virus shedding, as shown by positive PCR, gathered between 2 to 10 weeks in the different repetitions. Fecal samples analyzed at the end of the 12-week monitoring period by qPCR in a commercial diagnostic laboratory were consistent with the results of in house qPCR with one exception. Results differed in one sample in repetition A, where the commercial diagnostic laboratory detected a positive result not detected with the in-house qPCR.

#### Cage swabs and pooled fecal samples of the negative colony

No spread of MuAstV infection within the negative colony was detected based on cage swab results and fecal samples of the first five repetitions (A–E) (Fig 2). At the end of repetition F, MuAstV-infected animals (3 cages) were detected in the negative colony. This infection was detected by the pooled fecal samples. Cage swabs showed negative results. Infected mice were replaced by uninfected mice and the status of the negative colony was confirmed at the baseline test of the subsequent repetition G. However, at the end of repetition G, one cage was positive for MuAstV by feces PCR and cage swabs. As a consequence, the exact MuAstV prevalence of repetitions F and G are not known. Reasons for MuAstV transfer could be improper handling of cages while cage changing procedures.

#### Exhaust air particle PCR

In terms of EAP-PCR, each experimental repetition was evaluated positive and finished when two consecutive EAP results were positive for MuAstV. Despite low prevalence, MuAstV was detected in the gauze pieces mounted to the air handling unit prefilter already in week 1 and in week 2 after introducing positive mice to the test rack in 6 out of 7 experimental repetitions (Fig 2). As an exception, in repetition D, MuAstV was first detected after 4 weeks of exposure.

### Comparison of EAP-PCR and SBS for detecting *Myocoptes musculinus* infections in IVC-housed mouse colonies

#### Baseline tests

**Fig 3:**
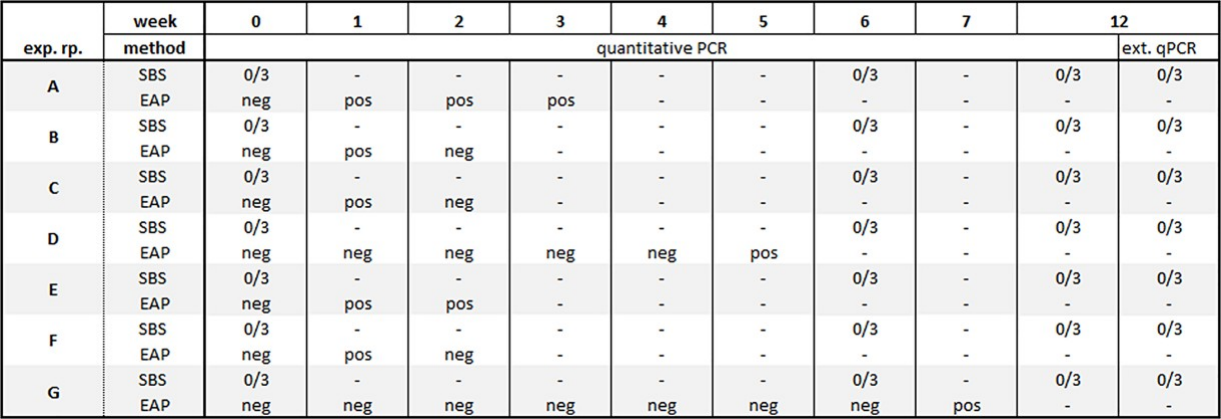
Comparison of qPCR results for soiled-bedding sentinels (SBS) and Exhaust Air Particle (EAP) monitoring at known *M*. *musculinus* prevalence. *M*. *musculinus* = *Myocoptes musculinus;* Exp. rp. = Experimental repetition; SBS = Soiled bedding sentinels; EAP = Exhaust air particle; ext. qPCR = quantitative PCR performed at an external provider; pos = positive EAP qPCR result for *M*. *musculinus*; neg = negative EAP qPCR result for *M*. *musculinus*; numerals = number of positive Sentinels per number sentinels tested for *M*. *musculinus* qPCR or serology dash, not tested; asterisk, samples between 0–1 copy / μL.

At the start of each of seven experimental repetitions, all SBS were negative for *M*. *musculinus* by qPCR of individual fur swabs of all SBS. No residual nucleic acids (*M*. *musculinus* DNA) were detected in the IVC-rack by qPCR analysis of gauze pieces exposed to the rack with the negative colony for one week. In summary, all baseline tests were negative (Fig 3).

#### Soiled bedding sentinels and EAP-PCR

In all 7 repetitions, no *M*. *musculinus* infestation of SBS was detected after 6 and 12 weeks of exposure time as shown by negative qPCR of fur swabs. In contrast, *M*. *musculinus* DNA was detected by EAP-PCR of gauze pieces mounted to the air handling unit prefilter in each repetition, in 5 out of 7 repetitions already after one week of exposure (Fig 3). As an exception, in repetition D and G, the first positive EAP-PCR test occurred after 5 and 7 weeks of exposure, respectively. As the main focus of the study was MuAstV detection, in repetition B, C, D, F and G no second confirmatory result was obtained.

## Discussion

Environmental sampling becomes a more and more popular technology for the hygienic monitoring of large rodent colonies housed in IVC-cages. Due to its potential relevance for biomedical research and its high dissemination, this study evaluates detection methods for Murine Astrovirus. Environmental sampling (EAP-PCR) and classical monitoring with soiled bedding sentinels were compared. Detection of various other organisms by EAP-PCR in such colonies proved to be more reliable than SBS monitoring as demonstrated by various studies [28,29,31,32,35–37,40]. In addition, a potential reduction of the number of sentinel mice contributes to the 3Rs. [41]

Our comparison showed that detection of MuAstV infections at low prevalence is possible with both methods, yet at different reliability. Compton et al. showed this already in a smaller scale study using a different rack system and a different sampling location. [27] Our study showed that the detection by EAP-PCR is faster, more sensitive and more reliable compared to SBS. Relying only on SBS serology, MuAstV infection of few cages of the colony would have been overlooked in 2 of 7 instances. By analyzing EAP samples, MuAstV was detected reliably (7/7) and quickly (in 6/7 rounds already in week 1). An equally high detection rate (7/7) by EAP-PCR was also found for our reference organism *M*. *musculinus*, detected in 5/7 repetitions already in week 1. *M*. *musculinus* was not detected at all by SBS, even though fur swabs were examined by qPCR analysis, a method with high sensitivity compared to the standard method microscopy. These results are consistent with those of another group which found soiled bedding unsuitable as a vector for the transfer of *M*. *musculinus* [33,34]. But the transfer of M. musculinus via soiled bedding is controversially discussed in literature. Ricart Arbona et al. [42] have demonstrated successful detection of *M*. *musculinus* via soiled bedding sentinels. In this study, transfer of only 2.5% dirty bedding of *M*. *musculinus* infected were sufficient to cause an infestation of sentinel mice. Other studies showed that the transfer of mites via soiled bedding to sentinels is rather unlikely, probably because it is vital for the mites to stay in contact to the host and transfer of mites only happens via direct contact of mice. [33,43] Inconsistent transmission of *M*. *musculinus* via dirty bedding may be caused by factors that are very variable. For example, may the size of the monitored colony and the amount of transferred bedding play a major role, as well as proportion of affected cages.

Mite infestation can be well treated with ivermectin. [43–45] But it should be considered that remaining DNA in the form of mite eggs or debris can lead to potentially false positive PCR results for weeks after treatment. In these cases, classical detection methods for *M*. *musculinus* such as tape test or fur pluck analysis, can represent further useful diagnostic tools. [46,47] Very low copy numbers were obtained when using EAP-PCR for the detection of the two pathogens (MuAstV 1–10 copies/μL, *M*. *musculinus* 1–15 copies/μL). Due to suitable qPCR amplification curves and proper controls (no-template-control (NTC) and negative control) these signals have been interpreted as clear positive results. The low copy numbers are presumably a consequence of the low cage prevalence set by the study design. For *M*. *musculinus*, cage prevalence was not more than 1 cage with 5 infested animals each, which was intended to simulate a hygienic break at the very beginning. In addition, it is known that mite density can decrease when infested animals grow older [48]. An exact quantification of the mite load on a given animal is currently not possible. This may also explain why we did not obtain second confirmatory EAP-PCR results in repetition B, C, D, F and G. In the last repetition (G), EAP-PCR samples were collected until positive results for both agents were detectable. A positive *M*. *musculinus* EAP-PCR result was obtained in week 7. This delayed detection may also be explained by the reasons given above. Experimental repetition D is an outlier in respect of the delayed EAP-PCR detection of both pathogens. Reasons for this phenomenon remain speculative but several paramount reasons have been ruled out. Accurate arrangement of all air conducting elements of the IVC Rack has been proven. Supply and exhaust air valves of the agent-positive cage positions have been checked correctly. MuAstV positive colony mice have been tested weekly for excretion via qPCR of fecal pellets and at least 10/15 positive mice were constantly shedding virus during the whole experimental period. MuAstV was inadvertently transferred to the negative colony in the last two experimental repetitions (F, G). MuAstV prevalence differed therefore in these rounds at a known amount. Since the status of the negative colony was only tested once per experimental repetition by qPCR analysis of fecal pellets (10 pellets/cage) and cage swabs of all cages, the time point of increased prevalence is not known. MuAstV infection of the negative colony at the end of round F (week 12) was detected by analysis of 10 pooled fecal pellets per cage; the cage swabs were negative. This indicates a very low virus load in the cage, suggesting the beginning of an infection. The MuAstV infection in experimental repetition G was detected by both sample types. We assume that the reason for MuAstV transfer was inappropriate handling of mice while cage changing procedures.

One further unexpected result is the missing MuAstV seroconversion of the SBS in round D despite positive qPCR findings (3/3) in the feces of the sentinels in week 10 and 12. These sentinels did not seroconvert, not even months later. Probably, the positive fecal PCR results originate from non-infectious (“dead”) virus particles shedded by infected colony animals, ingested by the sentinels and passed through the digestive tract without infection. Such passage normally does not provoke an antibody response. Therefore, when using SBS for the detection of MuAstV, it should be noted that false negative serological results may occur. The additional examination of fecal pellets of SBS might bring more safety here; however, this should be carried out at short intervals due to the limited duration of shedding.

A limitation of our study is that results cannot be generalized to all sorts of IVC systems. EAP-PCR as monitoring method should always be adapted and evaluated to the IVC rack system in use.

Furthermore, this experiment did not investigate the efficiency of detection with an even lower prevalence. Given the low copy numbers reached, it might be difficult to detect an infection, once the detection limit of the PCR has been reached. However, this also applies to SBS monitoring.

In conclusion, MuAstV can be detected by both EAP-PCR and SBS. In our opinion, EAP is the better detection method, because it is more reliable. *M*. *musculinus* was only detected by EAP-PCR. Therefore, environmental samples are the method of choice for its detection. Relying on SBS harbors a big risk of missing existing infestations.
